# Total arch replacement with bilateral extra-anatomical axillary artery bypass and frozen elephant trunk technique for Kommerell’s diverticulum

**DOI:** 10.1093/jscr/rjz211

**Published:** 2019-07-11

**Authors:** Daisuke Takeyoshi, Hiroto Kitahara, Takamitsu Tatsukawa, Daita Kobayashi, Hayato Ise, Sentaro Nakanishi, Taro Kanamori, Natsuya Ishikawa, Hiroyuki Kamiya

**Affiliations:** Department of Cardiac Surgery, Asahikawa Medical University, Hokkaido, Japan

## Abstract

A Kommerell’s diverticulum is a rare congenital aortic arch anomaly associated with a high rate of aortic rupture or dissection. Therefore, surgical or endovascular repair should be considered early. A 64-year-old man was incidentally found to have an aortic arch anomaly, Kommerell’s diverticulum, with a right aberrant subclavian artery and distal arch aneurysm. Hybrid total arch replacement with bilateral extra-anatomical axillary artery bypass and frozen elephant trunk technique was performed. This particular surgical approach would be a treatment option for any type of Kommerell’s diverticulum.

## INTRODUCTION

A Kommerell’s diverticulum (KD) with an aberrant right subclavian artery (ARSA) is a rare aortic arch anomaly. It can be associated with several complications, such as aortic aneurysm, distal embolization, compression of neighboring structures, and the most life-threatening complications, aortic dissection and rupture [1]. There are several reports describing the treatment strategies for KD [1–5]. However, which strategy is the best for KD remains unclear, because of its rarity. A case of hybrid total arch replacement (TAR) with bilateral extra-anatomical axillary artery bypass and frozen elephant trunk (FET) technique for KD is reported.

## CASE REPORT

**Figure 1:**
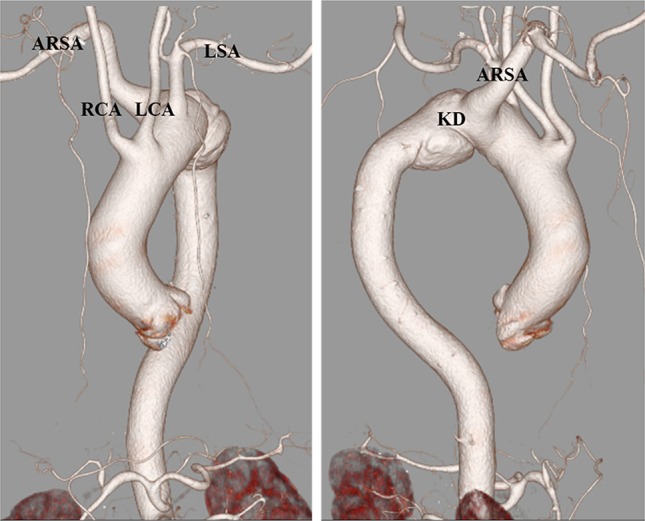
Preoperative computed tomography angiography. The Kommerell’s diverticulum with aberrant right subclavian artery. ARSA, aberrant left subclavian artery; KD, Kommerell’s diverticulum; LCA, left common carotid artery; RCA, right common carotid artery; LSA, right subclavian artery.

A 64-year-old man with cholelithiasis was referred to our institution. Then, he was incidentally found to have an aortic arch anomaly, KD with an ARSA (orifice diameter of 30 mm) and a distal arch aneurysm (diameter of 55 mm) (Fig. 1). Anatomically it seemed to be difficult to expose and directly manipulate the orifice of the ARSA. In our institution, extra-anatomical subclavian artery bypass is routinely performed in TAR to simplify the surgical technique [6]. We decided to proceed to hybrid TAR with bilateral extra-anatomical axillary artery bypass (adding extra-anatomical right axillary artery bypass) to avoid a deep and difficult end-to-end anastomosis of the ARSA.

Initially, two pieces of branch grafts (9 mm), cut from a four-branch graft (28 mm J-Graft; Japan Lifeline Co., Ltd., Tokyo, Japan), were anastomosed to both the left and the right axillary arteries and connected to selective cerebral perfusion (SCP) circuit. Median sternotomy was performed. The ARSA was deeply located at just the right side of the main bronchus. Cardiopulmonary bypass (CPB) was initiated via the ascending aorta and the superior and inferior venae cavae. A left ventricle vent was inserted from the right superior pulmonary vein during systemic cooling. The ascending aorta was clamped, and cardiac arrest was achieved with antegrade cardioplegic solution. The proximal anastomosis was performed using a 4–0 prolene. When the temperature reached 28°C, hypothermic circulatory arrest was started with retrograde cerebral perfusion (RCP), which not only to protect cerebral but also to flush atheromatous debris out from the arch vessels. side the aorta, there were many red-colored thrombi in the orifice of the ARSA. The ARSA was ligated just at the right side of the main bronchus. The left subclavian artery was ligated at the region of origin. SCP cannulas were inserted to bilateral common carotid arteries. Then, RCP was stopped, and SCP was initiated through both axillary artery grafts and selective cannulas to the right and the left common carotid arteries. The aorta was transected just distal to the left common carotid artery, and a FET, (J Graft FROZENIX® Japan Lifeline Co., Ltd.), was inserted antegradely and deployed. A stump of the graft was anastomosed to the four-branch graft by a 4-0 prolene. The CPB was re-started through the side branch of the four-branch graft. Next, distal and proximal grafts were anastomosed by a 4-0 prolene. Both axillary artery grafts were delivered into the mediastinal space passing through the intercostal space. The anastomoses of the first, second and third side branches of the graft to the right common carotid artery, the left common carotid artery, and the left axillary artery graft, respectively, were performed. Finally, the right axillary artery graft was anastomosed to the first side branch using end-to-side anastomosis. After complete re-warming, CPB was weaned uneventfully. The perfusion times were as follows: CPB (145 minutes), RCP (1 minute), and SCP (87 minutes).

**Figure 2:**
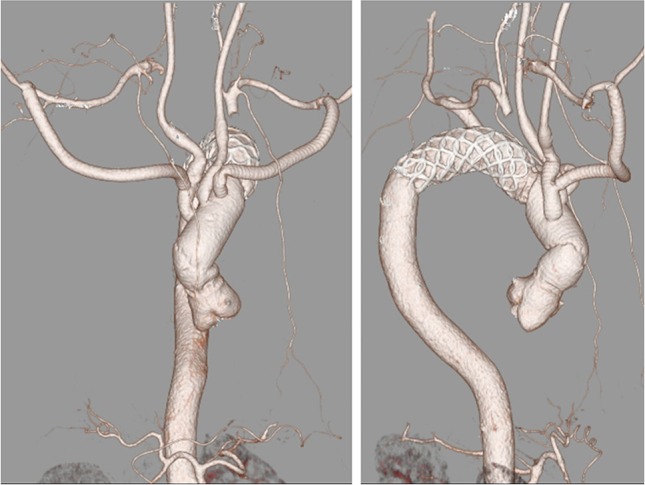
Postoperative computed tomography angiography.

The patient was transferred to the intensive care unit in stable condition. His postoperative course was uneventful, and he recovered without any neurological deficits. Postoperative CT showed no anastomotic stenosis or endoleak or kinking of the graft (Fig. 2).

## DISCUSSION

A KD with ARSA is a rare congenital anomaly due to a developmental error that results in a remnant fourth dorsal aortic arch. A KD carries a high risk of aortic dissection or rupture. This may be due to cystic medial necrosis often found in the diverticulum wall. Considering recent advancements and the excellent outcomes of surgical and/or endovascular treatment for KD, aggressive intervention is recommended when the diameter of the diverticulum orifice exceeds over 30 mm, and/or the diameter of the descending aorta adjacent to the diverticulum exceeds over 50 mm [1].

Currently, the best surgical technique for KD has not been determined. This can be attributed to the rarity of the disease. There have been a few reports published regarding surgical treatment strategies for KD. Kamiya and colleagues reported three surgical techniques for the treatment of KD [3]. In that article, complete anatomical repair of KD was achieved through the combination of the supraclavicular approach and left posterolateral thoracotomy. Ren *et al.* described a case of conventional TAR with FET technique [4]. Similarly Leone and colleagues reported the FET technique via median sternotomy in two patients with KD [5]. Idrees *et al.* described three different surgical strategies for KD, FET technique with additive endovascular completion for extensive disease of the distal thoracic aorta, FET technique for right-sided aortic arch and retroesophageal left subclavian artery or type three complete vascular ring, and complete thoracic endovascular aortic repair with cervical debranching [7].

A TAR with FET has been increasingly used for KD, as the latest report by Horikawa *et al.* reported a case of single-stage TAR with FET for KD [8]. They performed extra-anatomical subclavian artery bypass for only an aberrant left subclavian artery of KD with a right-sided aortic arch.

The difference in the present case from previous reports was that extra-anatomical axillary artery bypass was performed bilaterally before median sternotomy. This technique allowed us to avoid a deep and difficult ARSA anastomosis and resulted in shorter operative time and lower risk of bleeding. Furthermore, these bypasses could be used for quick initiation of stable and reliable SCP. This technique could also be used for other types of KD.

A TAR with bilateral extra-anatomical axillary artery bypass and FET technique was successfully performed for KD. This surgical strategy could be an effective treatment option for any type of KD.
